# Mass and Heat Transport Assessment and Nanomaterial Liquid Flowing on a Rotating Cone: A Numerical Computing Approach

**DOI:** 10.3390/nano12101700

**Published:** 2022-05-16

**Authors:** Qusain Haider, Azad Hussain, Aysha Rehman, Ahmed Ashour, Ali Althobaiti

**Affiliations:** 1 Department of Mathematics, University of Gujrat, Gujrat 50700, Pakistan; azad.hussain@uog.edu.pk; 2 Engineering Mathematics and Physics Department, Faculty of Engineering and Technology, Future University in Egypt, New Cairo 11845, Egypt; ahmed.ashour@fue.edu.eg; 3 Department of Mathematics, College of Science, Taif University, P.O. Box 11099, Taif 21944, Saudi Arabia; aa.althobaiti@tu.edu.sa

**Keywords:** numerical solution, rotating cone, rheological fluid, boundary layer, heat dissipation, buoyancy force, thermophoresis, Brownian motion

## Abstract

In the present study, we explore the time-dependent convectional flow of a rheological nanofluid over a turning cone with the consolidated impacts of warmth and mass exchange. It has been shown that if the angular velocity at the free stream and the cone’s angular velocity differ inversely as a linear time function, a self-similar solution can be obtained. By applying sufficient approximation to the boundary layer, the managed conditions of movement, temperature, and nanoparticles are improved; afterward, the framework is changed to a non-dimensional framework utilizing proper comparability changes. A numerical solution for the obtained system of governing equations is achieved. The effect of different parameters on the velocity, temperature, and concentration profiles are discussed. Tangential velocity is observed to decrease with an increase in the Deborah number, whereas tangential velocity increases with increasing values of the angular velocity ratio, relaxation to the retardation time ratio, and buoyancy parameter. Expansion in the Prandtl number is noted to decrease the boundary layer temperature and thickness. The temperature is seen to decrease with an expansion in the parameters of lightness, thermophoresis parameter, and Brownian movement. It is discovered that the Nusselt number expands by expanding the lightness parameter and Prandtl number, whereas it increases by decreasing the Deborah number. We also noticed that the Sherwood number falls incrementally in Deborah and Prandtl numbers, but it upsurges with an increase in the buoyancy parameter.

## 1. Introduction

Due to their applications in neurosciences and industries, non-Newtonian fluid flow models have been extensively examined by numerous scientists [1]. Non-Newtonian fluid applications consist of food mixing, intestinal chyme motion, portrait, blood flow, liquid metal, mixture flow, polymer, mercury amalgam flow, and fissile fuel slurry [2,3]. Nadeem et al. [4] described the impact of radiation on the boundary layer flow of a Jeffery fluid. Non-Newtonian fluids have attracted attention in recent years; their extensive use in many building and mechanical procedures is considered their major attribute [5,6]. Non-Newtonian heat and mass transfer fluids are important in food processing, paper making, heavy oils, and grease lubrication. Because of its unique applications in the real world, the mixed convection phenomenon has fascinated most scientific experts [7]. Several uses include solar central receivers during emergency shutdowns that are exposed to wind currents, low-speed heat exchangers, and cooled nuclear reactors [8,9]. Hering and Grosh [10] explored the consistently combined convection from a vertical cone for the small Prandtl number. Hassan et al. [11] studied the heat transportation of hybrid nanofluids under thermal radiation and Rosseland radiation over a rotating cone. Hassan et al. [12] elaborated a prescribed wall temperature case with magneto-hydrodynamic radiative hybrid particles. Arshad et al. [13] examined the comparative analysis of heat transfer of a nanostructured material over an exponentially stretching sheet. Arshad et al. [14] interpreted the Brownian and thermophoresis diffusion of a chemically reacting magneto-hydrodynamic nanofluid over an exponentially stretching sheet.

When nanoparticles are added to a fluid, they are collectively called a nanofluid. Nanofluids are nanoparticles dispersed in a base fluid. The nanoparticles used in nanofluids are regularly found in the form of metals, oxides, carbides, or carbon nanotubes. Masuda et al. [15] showed that different nanofluids (e.g., $Al_{2}O_{3}$-water, $SiO_{2}$-water, and $TiO_{2}$-water combinations) produced a $k_{nf}$ increase of less than 4.3% to 30% in volume fractions. Eastman et al. [16] introduced the phenomena of enhancing thermal conductivity for Cuo, $Al_{2}O_{3}$-water, and Cu-oil nanofluids. Nanofluid thermal conductivity was developed in the following decades as a function of several parameters.

In addition, Chamkha and Rashad [17] discussed time-dependent warmness and unsteady mass transmission from a rotating cone with chemical reaction and MHD combined, with free convectional flow and Soret and Dufour effects. The limit layer stream of non-Newtonian liquids in a permeable mass media, where gravity is the principal main impetus, has a wide scope of uses in design practice, especially in geophysics, geography, groundwater streams, and oil supply building. As a result of expansion in the generation of substantial amounts of unrefined oils and other materials whose flow behavior in shear rate cannot be portrayed by Newtonian connections, passable support of the archaeological characteristics of non-Newtonian liquid flow has become necessary. Consequently, a new phase in the development of fluid energy theory is underway.

Ellahi and Afzal [18] investigated the effects of temperature-dependent viscosity on a third-grade fluid in a porous channel. Saleem et al. [19] discussed unsteady fluid flow across a vertically spinning cone with neutral buoyancy and metallic subatomic effects. Rahman et al. [20] studied the impacts of nanoparticles and slip-on Jeffrey fluid through a mildly stenosed artery. Ahmed and Pop [21] investigated the flow of nanofluids from a given geometry filled with a porous medium during mixed convection. Hussain et al. [22] studied hybrid nanofluids with a mixed base over a rotating cone for heat transport investigation. Hussain et al. [23] introduced single-wall and multi-wall carbon nanotubes to study a thermal conductivity model for hybrid nanofluid. Hussain et al. [24,25] described time-dependent compressible fluid flow and heat transport enhancement over elliptical cylindrical obstacles placed in a rectangular cavity. Hussain et al. [26] introduced a two-phase flow over an embrittle cone for heat dissipation. Hussain et al. [27] investigated the enhancement of heat transfer in three-dimensional rotating flow over a cone. Hussain et al. [28,29,30,31] extensively examined three-dimensional rotating nano- and hybrid fluids over a linear and exponentially stretching surface with the externally applied effects of attractive field, warm air radiation, partial slip condition, and non-linear radiation. They found that hybrid nanofluids produce high-temperature allocation coefficients and reduced skin coefficients.

Rehman et al. [32] investigated the flow of pseudoplastic nanoliquids towards a versatile Riga sheet for assisting and opposing stagnation point flow. Hussain et al. [33] developed a computational model for the radiant energy kinetic molecular supposition of fluid-originated nanoparticle fluid in the presence of an induced magnetic force. Wróblewski performed experimental works on this research [34,35,36,37,38,39,40,41,42]. For time-dependent mathematical modeling, Saleem et al. [43] investigated a spinning cone in a viscoelastic fluid. Bahiraei et al. [44,45] proposed a heating element with new preemptive ladder-type helical baffles for a second law assessment on the flow of a nanofluid in a shell-and-tube heat exchanger, as well as a heat exchanger with new coreless inclined baffles using nanoparticle generation for thermal efficiency of a nanofluid in a shell-and-tube heat exchanger. Bahiraei et al. [46] discuss the tube heat exchanger operating and irreversibility characteristics of a mini shell with a nano liquid considering the impacts of nanoparticle shapes and fins.

**Figure 1:**
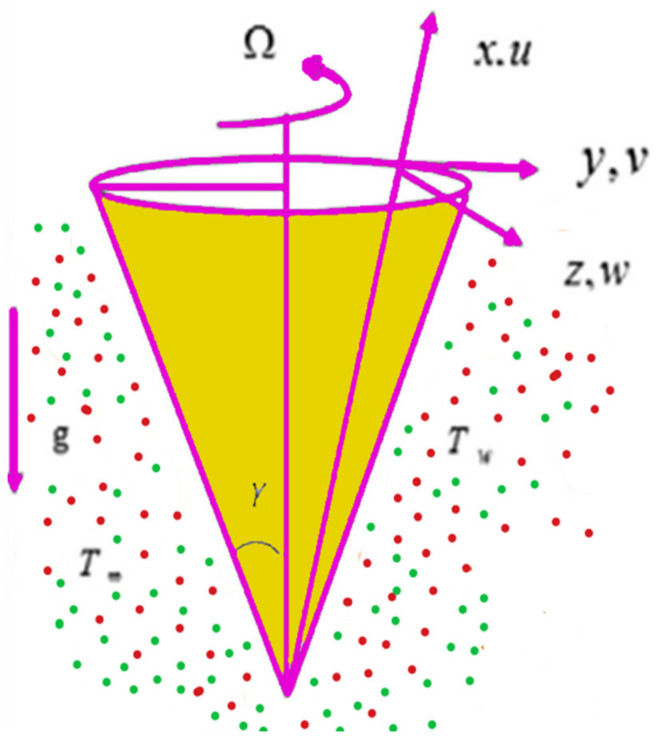
Graphical rotating cone structure.

The literature review reveals that, in general, researchers chose various effects to study the behavior of non-Newtonian fluids. To date, the characteristics of the Jeffery nanofluid model’s natural convection over a vertical rotating cone have not been studied; the novelty of the present work is to investigate the Jeffery nanofluid over a rotating vertical cone with thermophoresis and Brownian motion effects (Figure 1). The boundary layer approximation and similarity transformations are utilized to simplify the established model. A new efficient computational technique solves the governing coupled nonlinear system of equations. In addition, via the table, physical actions will be tested for the fluid’s industrial applications. The study’s outcomes are presented as graphs and tabulated data sets; moreover, a number of physical and engineering interests are also analyzed using distinct study parameters.

## 2. Mathematical Formulations

Let us consider the flow of an incompressible rheological nanofluid on a vertical spinning cone.

The Cauchy stress tensor of rheological fluid is [42],

(1) T=PI+S,

(2) $$S=\frac{\mu }{1+\lambda_{1}}\left(A_{1}+\frac{\lambda_{2}d}{dt}A_{1}\right),$$

(3) $$A_{1}=L+L^{t}.$$

where *P* represents the stress parameter, *I* is the identity matrix, $\lambda_{1}$ is the relaxation parameter, $\lambda_{2}$ is the retardation parameter, μ is the viscosity, and S is the extra stress tensor. V is the speed of the liquid, and $\frac{d}{dt}$ is the absolute change. This is the boundary surface equation of continuity, energy, concentration, and temperature for an uncompressible rheological fluid after employing the boundary layer theory and incorporating the gravity force, temperature, and concentration difference along with Brownian and thermophoresis diffusion.

The continuity equation, along with conservation of energy, momentum, and concentration equations, is given as follows [11,12,22].

(4) $$\frac{x\partial u}{\partial x}+\frac{x\partial w}{\partial z}+u=0,$$

(5) $$\begin{array}{c} \frac{\partial u}{\partial t}-\frac{v^{2}}{x}+\frac{u\partial u}{\partial x}+\frac{w\partial u}{\partial z}=\\ \frac{v}{1+\lambda_{1}}\left(\frac{\partial^{2}u}{\partial z^{2}}-\frac{v_{e}^{2}}{x}\right)+\frac{v\lambda_{2}}{1+\lambda_{1}}\left[\begin{array}{c} \frac{\partial w\partial^{2}u}{\partial z\partial z^{2}}+\frac{\partial^{2}u\partial u}{\partial x\partial z\partial z}\\ +\frac{w\partial^{3}u}{\partial z^{3}}+\frac{\partial^{3}u}{\partial z^{2}\partial t}+\frac{u\partial^{3}u}{\partial z^{2}\partial x} \end{array}\right]+\zeta^{*}gcos\alpha^{*}\left(C-C_{\infty }\right)\\ +g\zeta^{*}cos\alpha^{*}\left(T-T_{\infty }\right), \end{array}$$

(6) $$\begin{array}{c} \frac{\partial v}{\partial t}+\frac{uv}{x}+\frac{w\partial v}{\partial z}+\frac{u\partial v}{\partial x}=\\ \frac{v\lambda_{2}}{1+\lambda_{1}}\left[\frac{w\partial^{3}v}{\partial z^{3}}+\frac{\partial w\partial^{2}}{\partial z\partial z^{2}}+\frac{\partial^{3}v}{\partial z^{2}\partial t}+\frac{\partial u\partial^{2}v}{\partial z\partial x\partial z}+\frac{u\partial^{3}v}{\partial z^{2}\partial x}\right]\\ +\frac{\partial v_{e}}{\partial t}+\frac{\frac{v}{1+\lambda_{1}}\partial^{2}v}{\partial z^{2}}, \end{array}$$

(7) $$\frac{\partial T}{\partial t}+\frac{w\partial T}{\partial z}+\frac{u\partial T}{\partial x}=\frac{\rho^{*}c_{p}^{*}}{\rho c_{p}}\left(D_{B}\phi_{z}T_{z}+\frac{D_{T}T_{z}{}^{2}}{T_{\infty }}\right)+\frac{\alpha \partial^{2}T}{\partial z^{2}},$$

(8) $$\frac{\partial C}{\partial t}+\frac{w\partial C}{\partial x}+\frac{u\partial C}{\partial x}=\frac{D_{B}\partial^{2}C}{\partial z^{2}}+D_{T}\nabla^{2}T.$$

In Equations (4)–(8), we see that u, v, and w are the components of velocity in xy−plane, T denotes temperature, C is concentration, gβcos is the attraction impact, K and D represent the heat and mass diffusivity, $\alpha^{*}$ is the cone’s semivertical rim, β and $\beta^{*}$ are the temperature and volumetric extension coefficients, $T_{\infty },\;C_{\infty }$ are the free stream flow temperature and concentration, ν is the kinematic thickness, and $v_{e}$ denotes the free flow velocity. The initial (IC) and boundary conditions [11,12] are as follows:

(9) $$\begin{array}{c} u\left(0,x,z\right)=u_{i}\left(x,z\right),\;w=w_{i},v=v_{i},\;C=C_{i},\;T=T_{i},\;u\left(t,0,z\right)=w=0,\;\\ v=\frac{\Omega_{1}xsin\alpha^{*}}{1-st^{*}},\;C=C_{w},\;T=T_{W}. \end{array}$$

Defining the following argument of wall temperature changes [11,12,22]:

(10) $$\begin{array}{c} \eta =\frac{{\left(x\Omega sin\alpha^{*}\right)}^{0.5}z}{\nu {\left(1-st^{*}\right)}^{0.5}},\;v_{e}=\frac{x\Omega_{2}sin\alpha^{*}}{1-st^{*}},\;\alpha =\frac{\Omega_{1}}{\Omega },\;t^{*}=\Omega sin\alpha^{*}t,\;\\ w=\frac{{\left(sin\alpha^{*}\right)}^{\frac{1}{2}}{\left(\nu \Omega \right)}^{\frac{1}{2}}f\left(\eta \right)}{{\left(1-st^{*}\right)}^{\frac{1}{2}}},\;T-T_{\infty }=\left(T_{W}-T_{\infty }\right)\theta \left(\eta \right),\;\\ C_{w}-C_{\infty }=\frac{\left(C_{0}-C_{\infty }\right)x}{L}{\left(1-st^{*}\right)}^{-2},\;u\left(t,x,z\right)=-\frac{2^{-1}sin\alpha^{*}f^{\prime }\Omega x}{1-st^{*}},\;\\ v=\Omega xsin\alpha^{*}{\left(1-st^{*}\right)}_{\;}^{\frac{1}{2}}g\left(\eta \right),\;T_{W}-T_{\infty }=\frac{\left(T_{0}-T_{\infty }\right)xL^{-1}}{{\left(1-st^{*}\right)}^{2}},\;\\ Gr_{1}=\frac{cos\alpha^{*}\left(T_{0}-T_{\infty }\right)g\beta L^{3}}{\nu^{2}},\;Ec=\frac{v^{2}}{\left(C_{0}-C_{w}\right)C_{P}},\;\gamma_{1}=\frac{Gr_{1}}{Re_{L}^{2}},\;\\ Re_{L}=sin\alpha^{*}\Omega L^{2}\nu^{-1},\;Pr=\frac{\nu }{\alpha }. \end{array}$$

where $N_{T}$ and $N_{B}$ are the Brownian moment and thermophoresis parameters, Ec is Eckert Number, A is Deborah number, and $\gamma_{1},\;\gamma_{2}$ are the buoyancy parameters. The continuity equation (4) is uniformly fulfilled, and Equations (5)–(8) convert to the following form by using boundary conditions for the PWT case:

(11) $$\begin{array}{c} \frac{1}{1+\lambda_{1}}f^{\prime \prime \prime }-\left(f+\frac{1}{2}s\eta \right)f^{\prime \prime }-2\left(g^{2}-{\left(1-\alpha_{1}\right)}^{2}\right)+\\ \left(\frac{1}{2}f^{\prime }-s\right)f^{\prime }-2\gamma_{1}\left(\theta +N_{1}\phi \right)+\frac{2A}{1+\lambda_{1}}(f^{\prime }f^{\prime \prime \prime }+s\eta f^{iv}-{f^{\prime \prime }}^{2}+2ff^{iv}+4sf^{\prime \prime \prime }=0, \end{array}$$

(12) $$\begin{array}{c} s\left(1-\alpha_{1}-g-\frac{1}{2}\eta g\right)+\frac{1}{1+\lambda_{1}}g^{\prime \prime \prime }-\left(fg^{\prime }-f^{\prime }g\right)-\frac{A}{1+\lambda_{1}}(2sg^{\prime \prime }+\frac{1}{2}s\eta g^{\prime \prime \prime }\\ +\frac{1}{2}g^{\prime \prime }f^{\prime }-\frac{1}{2}f^{\prime \prime }g+fg^{\prime \prime \prime }=0, \end{array}$$

(13) $$\begin{array}{c} \mathrm{Pr}(-\frac{1}{2}s\theta f^{\prime }+f\theta^{\prime }+2s\theta +\frac{1}{2}s\eta \theta^{\prime })-\frac{PrEc}{1+\lambda_{1}}[-\frac{1}{4}{f^{\prime \prime }}^{2}+A(\frac{3}{8}s{f^{\prime \prime }}^{2}+\frac{1}{8}s\eta f^{\prime \prime \prime \prime }-\\ \frac{1}{8}{f^{\prime \prime }}^{2}+\frac{1}{4}ff^{\prime \prime }f^{\prime \prime \prime }-\theta^{\prime \prime }-N_{B}\psi^{\prime }\theta^{\prime }-N_{T}{\theta^{\prime }}^{2}=0 \end{array}$$

(14) $$\begin{array}{cc} Pr(2s\psi +\frac{1}{2}s\eta \psi^{\prime } & -\frac{1}{2}\psi f^{\prime }+\psi^{\prime }f\\ & -\psi^{\prime \prime }\frac{N_{T}}{N_{B}}[\mathrm{Pr}\left(-\frac{1}{2}s\theta f^{\prime }+f\theta^{\prime }+2s\theta +\frac{1}{2}s\eta \theta^{\prime }\right)\\ & -\frac{PrEc}{1+\lambda_{1}}[-\frac{1}{4}{f^{\prime \prime }}^{2}+A(\frac{3}{8}s{f^{\prime \prime }}^{2}+\frac{1}{8}s\eta f^{\prime \prime \prime }-\frac{1}{8}{f^{\prime \prime }}^{2}f\\ & +\frac{1}{4}ff^{\prime \prime }f^{\prime \prime \prime }-\theta^{\prime \prime }-N_{B}\psi^{\prime }\theta^{\prime }-N_{T}{\theta^{\prime }}^{2}=0, \end{array}$$

Now, the boundary conditions are

(15) $$f\left(0\right)=0,\;g\left(0\right)=\alpha_{1},\;f^{\prime }\left(0\right)=0,\;\theta \left(\infty \right)=0,\;\theta \left(0\right)=1,\;\psi \left(0\right)=1,\;\;\;f^{\prime }\left(\infty \right)=0,\;\psi \left(\infty \right)=0,\;g\left(\infty \right)=1-\alpha_{1},\;\theta \left(\infty \right)=0.\;$$

where $\alpha_{1}\;$is the cone and fluid velocity. When $\alpha_{1}=0,$ the fluid turns, and the cone is very quiet. For $\alpha_{1}=0.5,\;$the cone and the water are moving at precisely the same speed. On account of $\alpha_{1}=1,$ the liquid is very still, and the cone is pivoting. s is the temperamental parameter. If s is positive, it will assist the flow, and if s is negative, it will oppose the flow. $N_{1}$ is the proportion of the Grashof number.

In digressive and azimuthal directions, the skin friction coefficients for the PWT case are given [46]:

(16) $$C_{fx}=-\frac{2\mu {\left[\frac{\partial u}{\partial z}+\lambda_{2}\left(\frac{u\partial^{2}u}{\partial x\partial z}+\frac{\partial^{2}u}{\partial t\partial z}+\frac{w\partial^{2}u}{\partial z^{2}}\right)\right]}_{z=0}}{\rho \left(1+\lambda_{1}\right){\left[\Omega xsin\alpha^{*}\right]}^{2}{\left(1-st^{*}\right)}^{-2}},$$

(17) $$C_{fy}=\frac{2\mu {\left[{\Omega \mathrm{xsin}\alpha }^{*}{\left(1-st^{*}\right)}^{-1}\right]}^{-2}{\left[\lambda_{2}\left(\frac{u\partial^{2}v}{\partial x\partial z}+\frac{\partial^{2}v}{\partial t\partial z}+\frac{w\partial^{2}v}{\partial^{2}z}\right)+\frac{\partial v}{\partial z}\right]}_{z=0\;}}{\rho \left(1+\lambda_{1}\right)}.\;ambda\_\;flui$$

Now, after the transformations of these equations, we obtain:

(18) $$C_{fx}Re_{x}^{\frac{1}{2}}=\frac{1}{1+\lambda_{1}}{\left[-f^{\prime \prime }+\frac{A}{2}\left(f^{\prime }f^{\prime \prime }-3sf^{\prime \prime }+2ff^{\prime \prime }+\eta sf^{\prime \prime \prime }\right)\right]}_{\eta =0},$$

(19) $$C_{fx}Re_{x}^{\frac{1}{2}}=\frac{1}{1+\lambda_{1}}{\left[-f^{\prime \prime }+\frac{A}{2}\left(f^{\prime }f^{\prime \prime }-3sf^{\prime \prime }+2ff^{\prime \prime }+\eta sf^{\prime \prime \prime }\right)\right]}_{\eta =0},$$

Therefore, after using the dimensionless parameters, the Nusselt Number and Sherwood Number are given as follows:

(20) $$Nu_{x}Re_{x}^{\frac{1}{2}}=-\theta^{\prime }\left(0\right),$$

(21) $$Sh_{x}Re_{x}^{\frac{1}{2}}=-\psi^{\prime }\left(0\right).$$

Now, the Reynolds number is:

(22) $$Re_{x}=\frac{x^{2}\Omega sin\alpha^{*}{\left(1-st^{*}\right)}^{-1}}{\upsilon }.$$

## 3. Numerical Solution

The second-order nonlinear partial differential equations (number of independent variables) are converted to ordinary differential equations (single independent variable) with the assistance of appropriate transformations. Together with the numerical analysis with boundary conditions, we used the shooting technique to find these equations. Equations (23)–(27) are solved by Maple and numerically.

**Figure 2:**
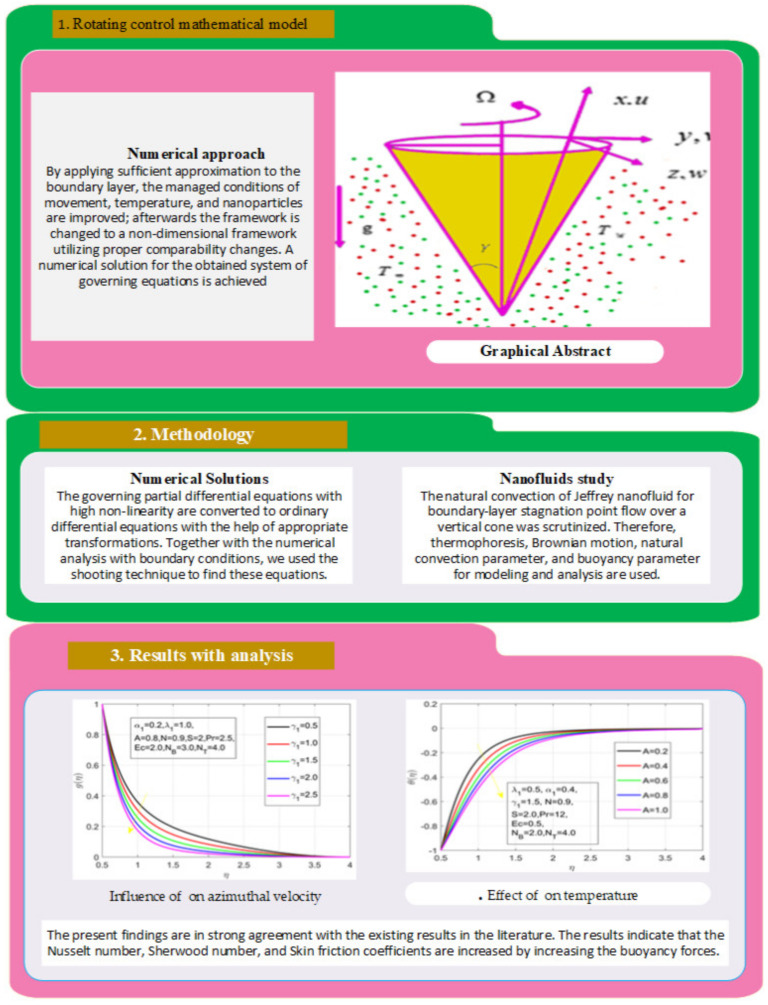
Workflow-based mathematical rotating cone flow nanofluid model using the numerical method.

We define new variables that simplify high-order differential equations into the first-order equation (Figure 2), i.e.,

(23) $$F=y_{1},\;F^{\prime }=y_{2},\;g=y_{5},\;F^{\prime \prime }=y_{3},\;F^{iv}=y_{4}^{\prime },\;g^{\prime }=y_{6},\;g^{\prime \prime }=y_{7}^{\prime },\;F^{\prime \prime \prime }=y_{4},\;\;\theta =y_{8},\;\theta^{\prime }=y_{9},\;\theta^{\prime \prime }=y_{9}^{\prime },\;\psi =y_{10},\;\psi^{\prime }=y_{11},\;\psi^{\prime \prime }=y_{11}^{\prime }.$$

Now, the new equations are

(24) $$\begin{array}{cc} y_{4}^{\prime }=\frac{1+\lambda_{1}}{A\left(\frac{1}{2}s\eta +y_{1}\right)} & [-\frac{y_{4}}{1+\lambda_{1}}+\left(y_{1}+\frac{1}{2}s\eta \right)y_{3}-\left(\frac{1}{2}y_{2}-s\right)y_{2}\\ & +2(y_{5}^{2}-{\left(1-\alpha_{1}\right)}^{2}+2\gamma_{1}\left(y_{8}+N_{1}y_{10}\right)\\ & -\frac{A}{\left(1+\lambda_{1}\right)}\left(\frac{1}{2}y_{2}y_{4}-\frac{1}{2}y_{3}^{2}+2sy_{4}\right)]=0, \end{array}$$

(25) $$y_{7}^{\prime }=\frac{1+\lambda_{1}}{A\left(\frac{1}{2}s\eta +y_{1}\right)}[-\frac{y_{4}}{1+\lambda_{1}}-\left(y_{1}y_{6}-y_{2}y_{5}\right)+s\left(1-\alpha_{1}-y_{5}-\frac{1}{2}\eta y_{6}\right)\;-\frac{A}{1+\lambda_{1}}\left(2sy_{7}+\frac{1}{2}y_{7}y_{2}-\frac{1}{2}y_{3}y_{5}\right)]=0,$$

(26) $$\begin{array}{cc} y_{9}^{\prime }=\mathrm{Pr}(2sy_{8}+ & \frac{1}{2}s\eta y_{9}-\frac{1}{2}sy_{8}y_{2}+y_{1}y_{9})\\ & -\frac{EcPr}{1+\lambda_{1}}[-\frac{1}{4}y_{3}^{\prime }\\ & +A(\frac{3}{8}sy_{3}^{2}+\frac{1}{8}s\eta y_{4}-\frac{1}{8}s\eta y_{4}-\frac{1}{8}y_{3}^{2}y_{1}+\frac{1}{4}y_{1}y_{3}y_{4}-N_{1}y_{11}y_{9}\\ & -N_{B}y_{9}^{2})]=0, \end{array}$$

(27) $$\begin{array}{cc} y_{11}^{\prime }=\mathrm{Pr}(2sy_{10} & +\frac{1}{2}s\eta y_{11}-\frac{1}{2}sy_{10}y_{2}+y_{1}y_{11}\\ & -\frac{N_{T}}{N_{B}}[\mathrm{Pr}(2sy_{9}+\frac{1}{2}s\eta y_{9}-\frac{1}{2}sy_{8}y_{2}+y_{1}y_{9})\\ & -\frac{EcPr}{1+\lambda_{1}}[-\frac{1}{4}y_{3}^{2}\\ & +A(\frac{3}{8}sy_{3}+\frac{1}{8}s\eta y_{4}-\frac{1}{8}s\eta y_{4}-\frac{1}{8}y_{3}^{2}y_{1}+\frac{1}{4}y_{1}y_{3}y_{4}\\ & -N_{B}y_{11}y_{9}-N_{T}y_{9}^{2})]]=0, \end{array}$$

Along with limitations

(28) $$y_{1}\left(0\right)=0,\;y_{5}\left(0\right)=\alpha_{1},\;y_{2}\left(0\right)=0,\;y_{8}\left(0\right)=y_{10}\left(0\right)=1,\;y_{8}\left(\infty \right)=0,\;y_{2}\left(\infty \right)=y_{10}\left(\infty \right)=0,\;y_{5}\left(\infty \right)=1-\alpha_{1}.$$

## 4. Discussion and Graphical Result

In this section, the impact of several study parameters, such as the rotation ratio, buoyancy ratio, Deborah number, and relaxation parameter, are presented on different profiles. These profiles include azimuthal g, and tangential velocity profiles $-f^{\prime }$, temperature θ, and concentration profile ϕ. The influence of distinct training parameters is also computed for quantities of physical interest, namely skin coefficients in the x and y direction, Nusselt $Nu_{x}$, and Sherwood number $Sh_{x}$. The achieved outcomes for quantities of engineering interest are presented in Table 1 and Table 2.

**Figure 3:**
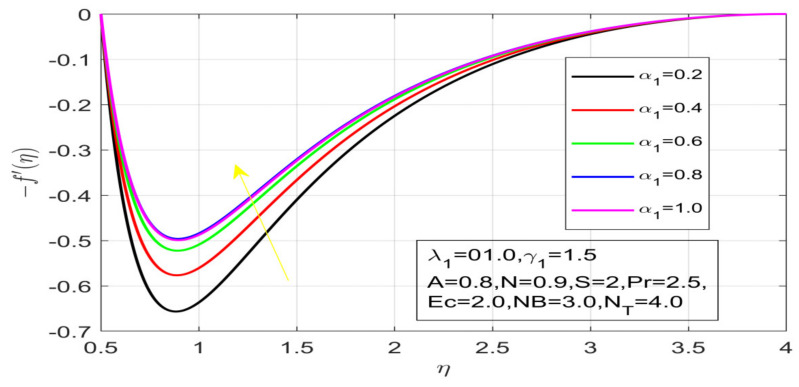
Influence of α_1 on the velocity distribution.

**Figure 4:**
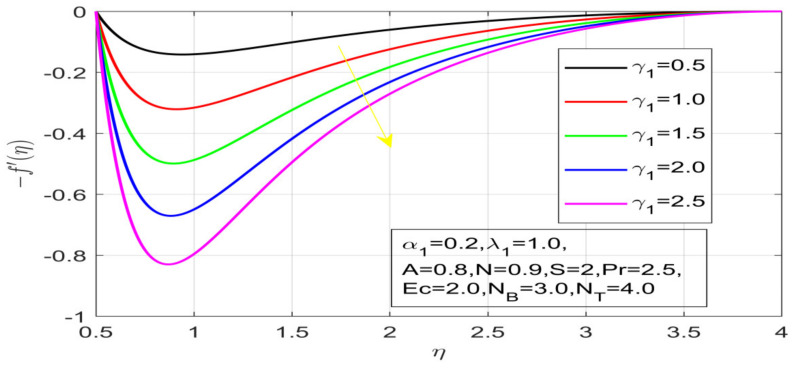
Influence of γ_1 on the velocity profile.

**Figure 5:**
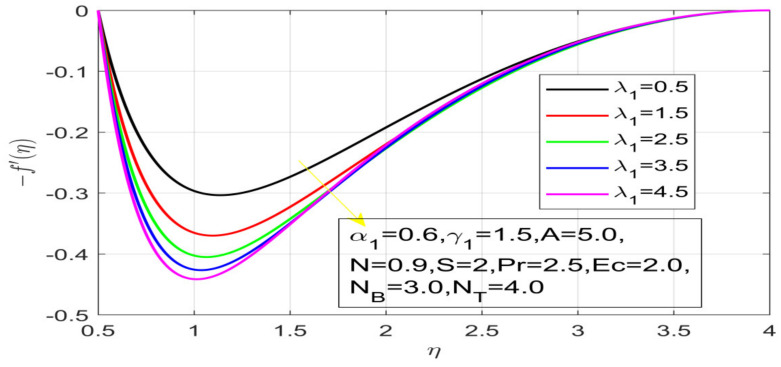
Influence of λ_1 on the velocity profile.

**Figure 6:**
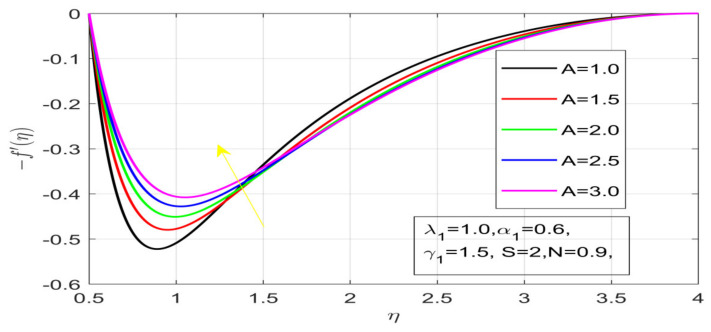
Impact of A on the velocity profile.

**Figure 7:**
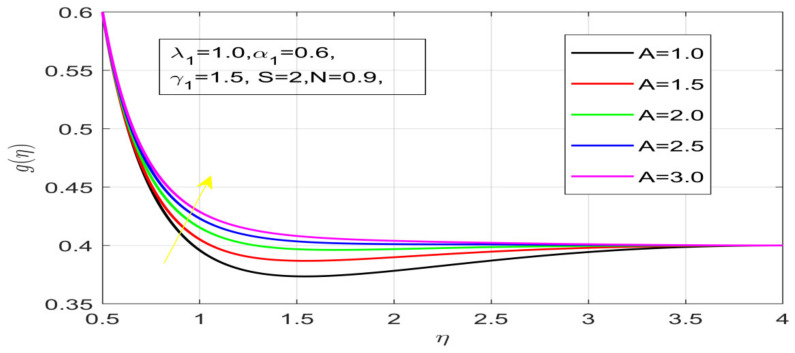
Effect of A on azimuthal velocity.

**Figure 8:**
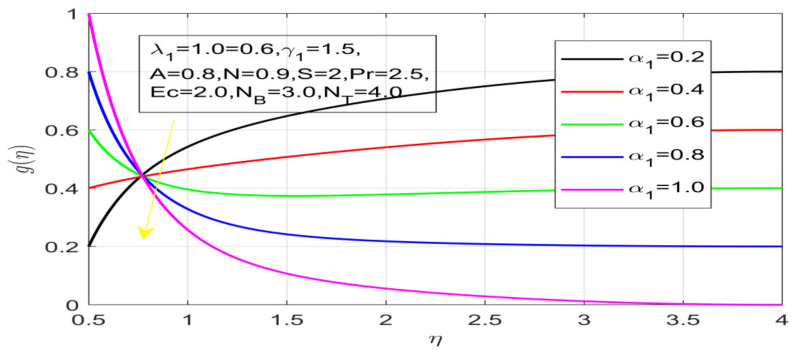
Influence of α_1 on azimuthal velocity.

**Figure 9:**
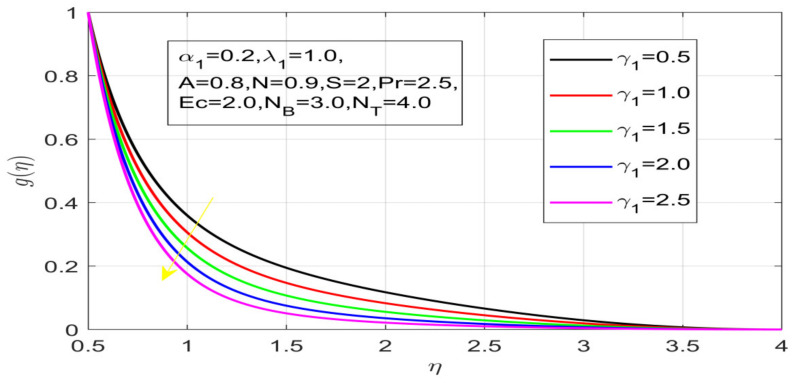
Influence of γ_1 on azimuthal velocity.

**Figure 10:**
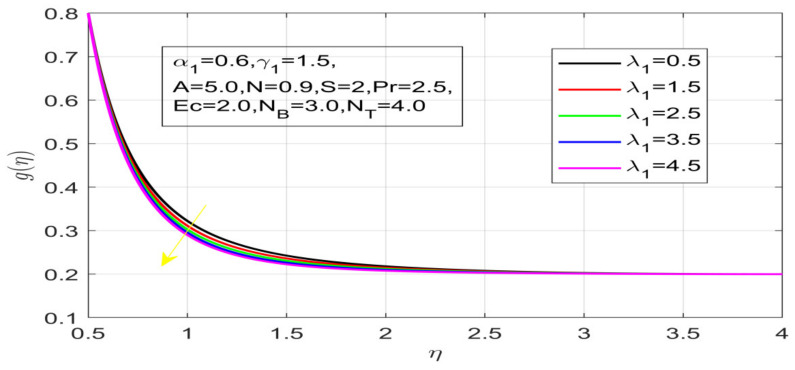
Impact of λ_1 on azimuthal velocity.

**Figure 11:**
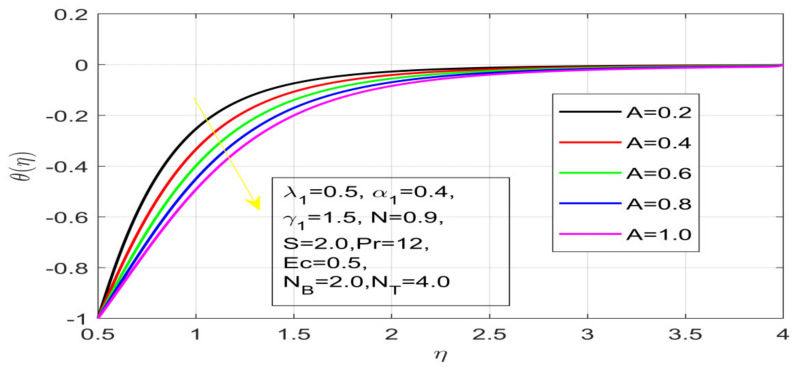
Effect of A on temperature.

**Figure 12:**
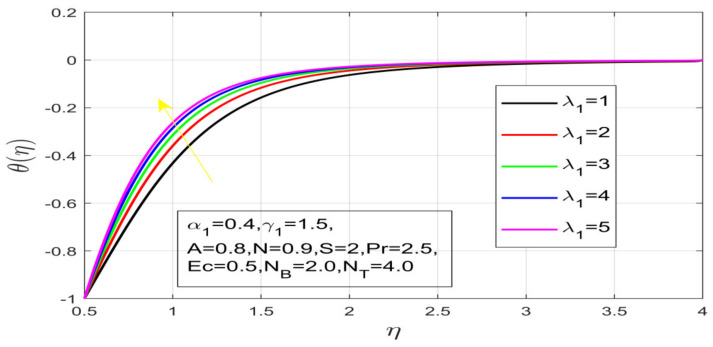
Impact on λ_1 on temperature.

**Figure 13:**
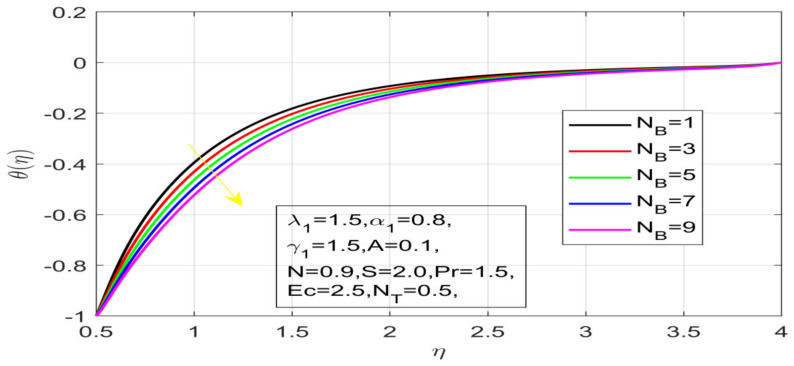
Influence of N_B on temperature.

**Figure 14:**
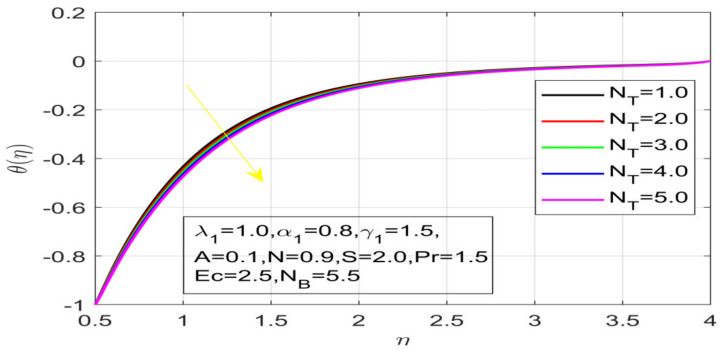
Variation in N_T on temperature.

**Figure 15:**
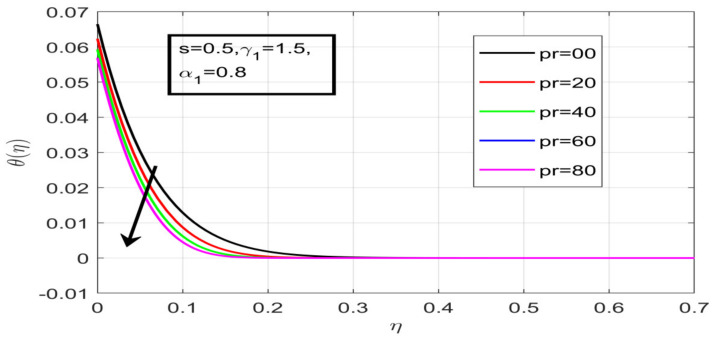
Contact of Pr on temperature.

**Figure 16:**
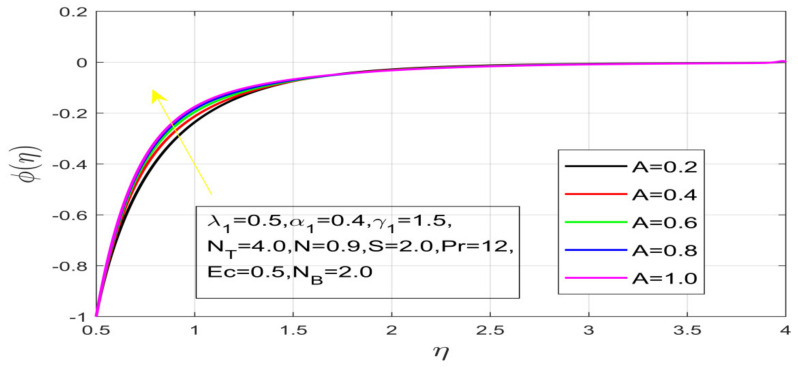
Influence of A on concentration.

**Figure 17:**
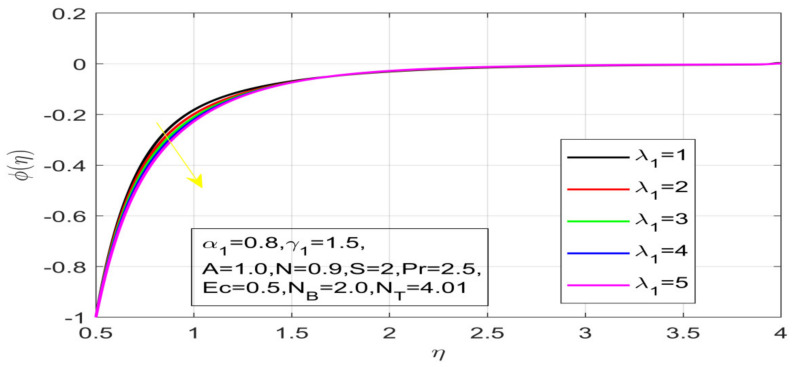
Contact of λ_1 on concentration.

**Figure 18:**
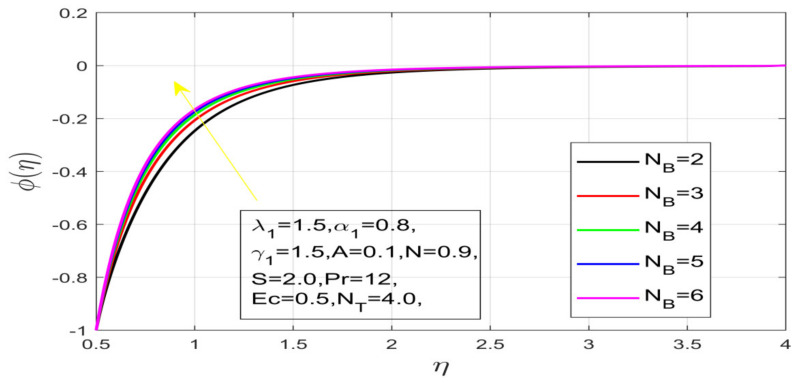
Impact of N_B on concentration.

**Figure 19:**
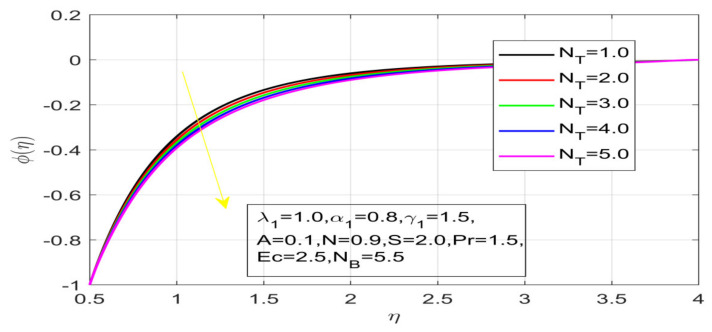
Impact of N_T on concentration.

**Figure 20:**
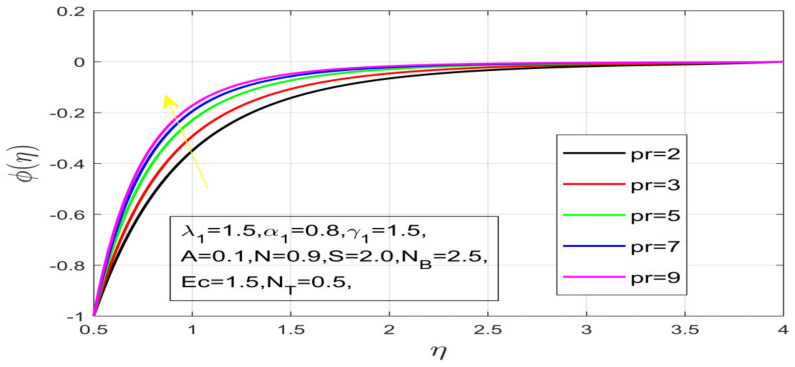
Variation in Pr on concentration.

**Figure 21:**
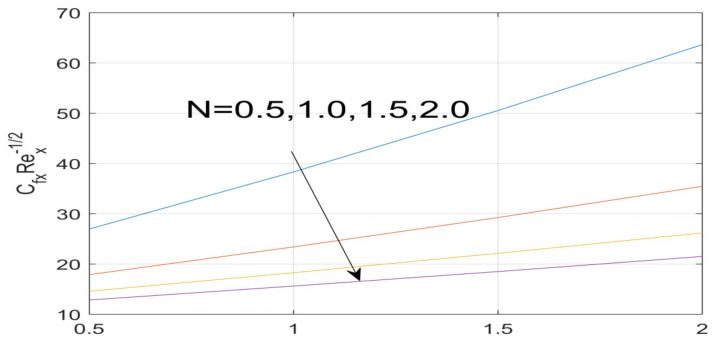
Impact of N on f^″ (0).

**Figure 22:**
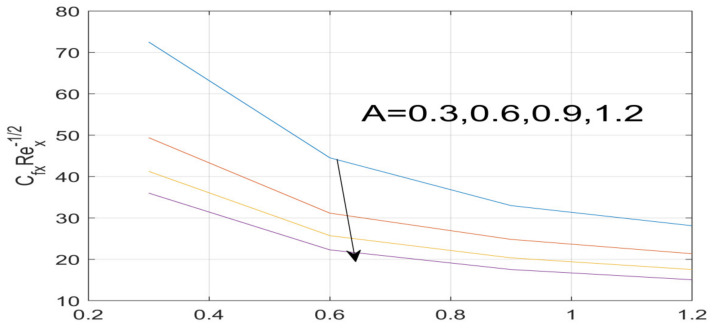
Effect of A on f^″ (0).

**Figure 23:**
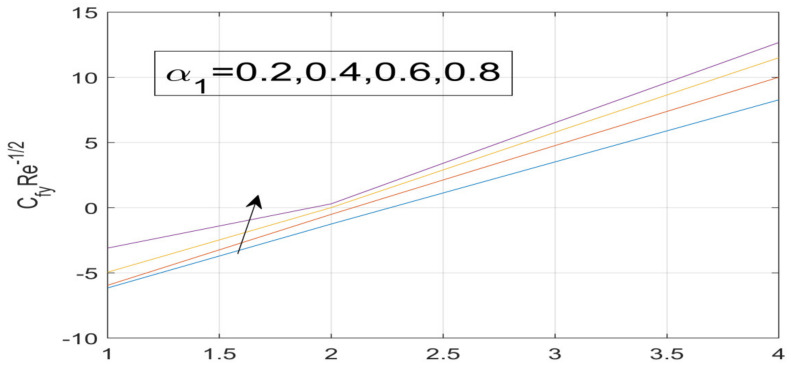
Influence of α_1 on g^′ (0).

**Figure 24:**
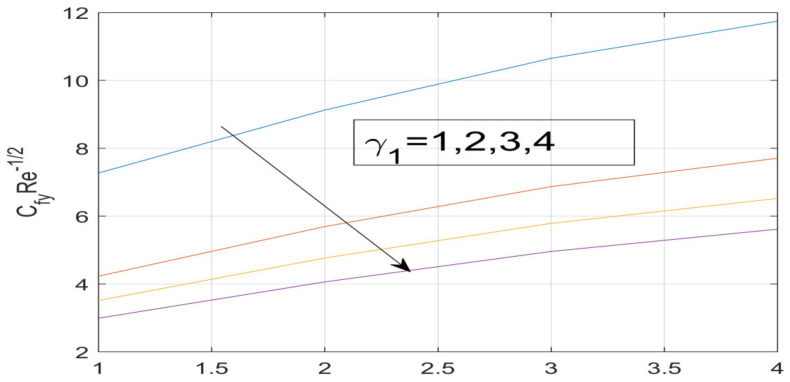
Variation in γ_1 on g^′ (0).

**Figure 25:**
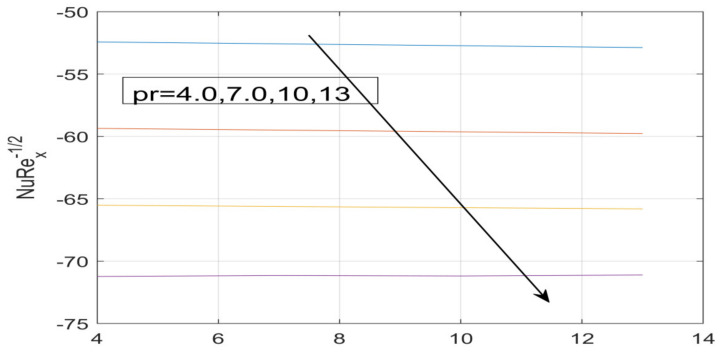
Effect of Pr on −θ^′ (0).

**Figure 26:**
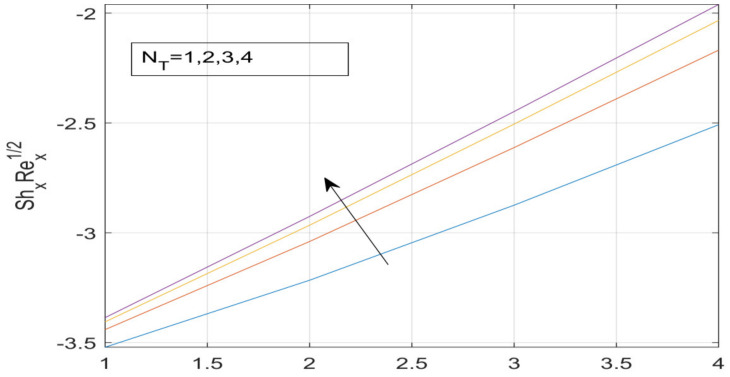
Effect of N_T of on −ψ^′ (0).

First, the supervisory conditions are rearranged using a change in closeness. Then, the decreased exceptionally nonlinear coupled differential equation is explained logically with the assistance of the numerical arrangement. Figure 1 describe the geometry of the problem. Figure 2 define the workflow of the problem. The results on the tangential velocity of $\alpha_{1},\;\;\lambda_{1},\;\;\gamma_{1}$, and Deborah number *A* are shown in Figure 3, Figure 4, Figure 5 and Figure 6. Tangential velocity decreases for A as it increases for all other parameters. Meanwhile, when $\gamma_{1}=0.5$, the liquid and the cone revolve similarly with good accurate velocity, and the movement is only due to the gradient of high pressure; for example, $\gamma_{1}=1$. When $\alpha_{1}=0.5$, the velocity profile increases. On the other hand, the velocity profile decreases for $\alpha_{1}<0.5$. Therefore, when $\alpha_{1}<0$ is detected, the velocity field at the edge of the boundary layer approaches an oscillatory form asymptotically. Figure 7, Figure 8, Figure 9 and Figure 10 show the variance of the Deborah number A, the angular velocity ratio $\alpha_{1}$, the buoyancy parameter $\gamma_{1}$ and $\lambda_{1}$, concerning azimuthal velocity g, respectively. The azimuthal velocity behavior contrasts with the tangential velocity behavior. It is observed that a rise in A and $N_{1}$ increases the azimuthal velocity, and that this behavior is contrary to that of $\lambda_{1}.$ The impact of Pr on velocity in the tangential direction is very small and the same as in the azimuthal direction; therefore, we are neglecting this profile. Figure 11, Figure 12, Figure 13, Figure 14 and Figure 15 present the impact of temperature for different values of $Pr,\;\;\lambda_{1},\;\;N_{T}\;and\;N_{B}.$ To increase the importance of Pr, spectral boundary layer strength is indicated to decrease. This is because there is a lower warm conductivity of higher Prandtl number liquid, creating a slender warm boundary layer. The temperature and density of the boundary layer decrease as the Prandtl number Pr increases; this reveals the fact that the thermal diffusivity changes correspondingly with an increase in Pr, which thus relates to less energy transfer capacity and a prompt decrease in the warm boundary layer and temperature profile. The temperature is seen to decrease with an expansion in $\lambda_{1}$, yet increases with an expansion in $N_{T}$ and $N_{B}$, the conduct of nanoparticles fixation C for different Pr, the lightness proportion, the thermophoresis parameter, and the parameters of Brownian movement. It is understood that the group of nanoparticles expanded the range of the thermophoresis parameter. The values of concentration ϕ decrease when the values of the *Pr* increase. The value of A increases, then the value of $N_{1}$ also increases due to the increase in these two values of skin friction, as shown in Figure 16, Figure 17, Figure 18, Figure 19 and Figure 20. Biologically, near the cone boundaries, we can say that the temperature of the fluid is less than the temperature of the wall, which eventually increases the $Gr_{1}$ relative to $Gr_{2}$, so greater $N_{1}$ gives greater values of skin friction. Figure 21 and Figure 22 show that the tangential skin friction coefficient increases respectively by increasing $N_{1}$ and A. Physically, near the cone borders, we can assume that the temperature of the surface is higher than the temperature of the substance, which eventually increases the $Gr_{2}$ as opposed to $r_{1}$, while greater $N_{1}$ gives greater values of skin friction. From Figure 23 and Figure 24, it is noted that the coefficients of $g\left(0\right)$ increase with the increase in $\gamma_{1}$ and $\alpha_{1}$, but the action is contrary for $\lambda_{1}$. As the impacts of Pr and Ec in tangential and azimuthal directions on the velocity profiles are relatively small, these profiles are thus ignored. Figure 25 and Figure 26 show that with increases in Pr, Ec, and $N_{T}$, the $Nu_{x}Re_{x}^{\frac{1}{2}}$ and $Sh_{x}Re_{x}^{\frac{1}{2}}$ also increase. The unrelated skin coefficients were found to decrease as $\lambda_{1}$. The Nusselt and the Sherwood number statistical figures number separately for different estimates of A, Pr, and $\lambda_{1}$. The quantity of Nusselt increases by expanding $\lambda_{1}$ and Pr and by decreasing the quantity of A. We noticed that the quantity of the Sherwood number exhibits a decreasing behavior for *A* and *Pr*, whereas it increases for an increase in $\lambda_{1}$, as shown in Table 1 and Table 2.

## 5. Conclusions

In this study, the Jeffery fluid under Brownian and thermophoresis diffusion are investigated over a vertically rotating cone. Therefore, thermophoresis, Brownian motion, natural convection parameter, and buoyancy parameter are used for modeling and analysis. The governing nonlinear coupled ODEs are then numerically solved using MATLAB bvp4c methodology. The present findings are compared and discovered to be in strong agreement with the existing results in the literature. The results indicate that the Nusselt number, Sherwood number, and Skin friction coefficients are increased by increasing the force of buoyancy. The buoyancy force interrupts the oscillations in the velocity that occurs due to excessive angular momentum convection. The analysis summary is as follows:

1) The increment in the rotation ratio, buoyancy ratio, relaxation parameter, and Deborah number $\alpha_{1},\;\lambda_{1},\;\gamma_{1}$, and A, respectively, increases the tangential velocity profile; as a result, the thickness of the momentum boundary layer increases.
2) The azimuthal velocity profile decays smoothly as the buoyancy ratio, relaxation parameter, and Deborah number are increased, whereas the thickness of the momentum layer disperses with augmentation in the rotation parameter.
3) The temperature profile increases as the Deborah number and relaxation parameter are incremented; this ultimately expands the thermal boundary of rotating flow.
4) The thermal boundary layer expanded with the upsurge in Brownian motion parameter and Prandtl number, whereas as the thermophoresis parameter is increased, contraction was observed in the thickness of the thermal boundary.
5) The thickness of the concentration layer decreased with an increment in Deborah number, relaxation parameter, Brownian motion parameter, and thermophoresis parameter.
6) The skin friction coefficient in the x-direction upsurges with an increment in the Deborah number, and it decays with an increasing buoyancy ratio. The rotation parameter enhances the skin coefficient in the y-direction, and with increasing buoyancy force, sinks in the y-direction.
7) Prandtl number augmentation shows a decline in the heat transfer coefficient, and mass coefficient upsurges abruptly with an increment in the thermophoresis parameter.

## Figures and Tables

**Table 1:** Variation in skin friction for different parameters.

A	$\lambda_{1}$	$\gamma_{1}$	$\alpha_{1}$	$Cf_{x}$	$Cf_{y}$
1.0	1.5	1.5	0.6	10.44303	−6.05803
1.5				7.40914	−6.79113
2.0				5.87893	−7.53710
2.5				4.98261	−8.49709
3.0				4.38739	−8.497092
	1.0	1.0		2.31903	−1.48343
0.9	1.5	1.5	0.2	2.61075	−1.51450
	0.2	0.2		2.847548	−1.573754
	2.5	2.5		3.027231	−1.709276
	3.0	3.0		3.15504	−1.709276
			0.2	4.63807	−2.96687
0.6	1.5	1.5	0.4	3.31065	−0.769717
			0.6	2.42189	1.35522
			0.8	1.985445	5.68020
			1.0	2.00452	5.68020
				4.63807	−2.96687
0.8	1.5	1.5	0.2	7.52467	−2.90302
				10.41603	−2.86442
				13.22616	−2.88062

**Table 2:** Variation in Sh and Nu for different parameters.

** A **	** $\lambda_{1}$ **	** $N_{B}$ **	$N_{T}$	Pr	$Nu_{x}$	$Sh_{x}$
0.8	0.5	2.5	0.5	2.0	−1.40971	−1.94585
				3.0	−1.28835	−2.65186
				5.0	−1.13464	−3.25699
				7.0	−1.03110	−3.68448
				9.0	−0.95847	−4.01456
			1.0		−0.88942	−2.01438
0.8	1.0	5.5	2.0	1.5	−0.86044	−1.98444
		3.0			0.83677	−1.95356
		4.0			−0.81697	−1.92151
		5.0			−0.80007	−1.88826
	1.0				−1.46261	1.25144
0.6	1.5	2.0	5.5	1.5	−1.18703	−0.92170
		3.0			−1.03336	−1.47372
		4.0			−0.92024	−1.70850
		5.0			−0.83076	−1.831024
1.0					−0.863587	−2.30070
1.5	1.5	3.0	4.0	2.5	−0.62539	−0.62539
	2.0				−0.963370	−2.25823
	2.5				−1.046995	−2.22162
	3.0				−1.11684	−2.1904618

## Data Availability

All data supporting this study are available in the article.

## Nomenclature

$\alpha_{1}$	Rotational ratio.	$Gr_{1},\;Gr_{2}$	Grashof number for PWT case.
$\nu_{e}$	Free stream velocity.	$\gamma_{1},\;\gamma_{2}$	Buoyancy parameters for PWT.
u,ν,w	Components of velocity in x, y, and z (ms^−1^).	$\alpha^{*}$	Angle of rotation.
T,C,D	Temperature, concentration, and mass diffusivity, respectively.	N	The ratio of Grashof number.
$\Omega_{1},\Omega_{2}$	Cone and fluid’s angular velocities, respectively.	$Re_{L}$	Reynolds number.
Ω	Sum of cone and fluid velocities.	Pr	Prandtl number.
$\beta ,\beta^{*}$	Volumetric coefficients of temperature and concentration expansion, respectively.	Sc	Schmidt or Sherwood number.
s	Unsteadiness in free stream velocity.	η	Similarity variable.
PWT	Prescribed wall temperature.	$T_{\infty },\;C_{\infty }$	Temperature and concentration of free stream.
*f,g*	Velocity profiles.	$k_{f}$	Base fluid’s thermal conductivity (Wm^−1^K^−1^).
θ,φ	Temperature and concentration profiles.	*PDE’s*	Partial Differential Equations.
*L*	Characteristic length.	*ODE’s*	Ordinary Differential Equations.
*BLA*	Boundary-Layer Approximation.	*BVP*	Boundary Value Problem.
ν	Dynamic viscosity (mPa).	$N_{B}$	Brownian Motion parameter.
$N_{T}$	Thermophoresis parameter.	$\lambda_{1}$	Relaxation parameter.
$\lambda_{2}$	Retardation parameter.	*MHD*	Magneto-Hydrodynamic.
$k_{nf}$	Thermal conductivity of nanofluid (Wm^−1^K^−1^).	ρ	Density of fluid (Kgm^−3^).
